# Can learning health systems help organisations deliver personalised care?

**DOI:** 10.1186/s12916-017-0935-0

**Published:** 2017-10-02

**Authors:** Bright I. Nwaru, Charles Friedman, John Halamka, Aziz Sheikh

**Affiliations:** 1 Krefting Research Centre, Department of Internal Medicine, University of Gothenburg, Gothenburg, Sweden; 2 Wallenberg Centre for Molecular and Translational Medicine, Institute of Medicine, University of Gothenburg, Gothenburg, Sweden; 3 Asthma UK Centre for Applied Research, Usher Institute of Population Health Sciences and Informatics, University of Edinburgh, Edinburgh, UK; 4 Department of Learning Health Sciences, University of Michigan, Ann Arbor, MI USA; 5 Beth Israel Deaconess Medical Center/Harvard Medical School, Boston, MA USA; 6 Brigham and Women’s Hospital/Harvard Medical School, Boston, MA USA; 7 Asthma UK Centre for Applied Research, Centre for Medical Informatics, Usher Institute of Population Health Sciences and Informatics, The University of Edinburgh, Teviot Place, Edinburgh, EH8 9AG UK

**Keywords:** Precision medicine, P4 medicine, Personalised medicine, Stratified medicine, Learning health system, Asthma

## Abstract

There is increasing international policy and clinical interest in developing learning health systems and delivering precision medicine, which it is hoped will help reduce variation in the quality and safety of care, improve efficiency, and lead to increasing the personalisation of healthcare. Although reliant on similar policies, informatics tools, and data science and implementation research capabilities, these two major initiatives have thus far largely progressed in parallel. In this opinion piece, we argue that they should be considered as complementary, synergistic initiatives whereby the creation of learning health systems infrastructure can support and catalyse the delivery of precision medicine that maximises the benefits and minimises the risks associated with treatments for individual patients. We illustrate this synergy by considering the example of treatments for asthma, which is now recognised as an umbrella term for a heterogeneous group of related conditions.

## Introduction

The Human Genome Project and the subsequent sequencing of the human genome dramatically redefined our understanding of disease processes, diagnosis, therapeutics, and prevention [1–4]. These advances laid the foundations for President Obama’s launch in 2015 of the Precision Medicine Initiative, which aims to integrate the vast and ever-increasing quantities of genomic, biological, health, administrative, environmental, and behavioural data on individuals in order to achieve more individually tailored decision making and personalised healthcare [5]. This Initiative has generated considerable enthusiasm, but it has also attracted some skepticism [6, 7]. There have been high profile demonstrations of the promise of precision medicine in, for example, cystic fibrosis and cancer [8–12]. The discovery and clearer understanding of the cystic fibrosis transmembrane conductance regular (*CFTR*) gene variants as the cause of cystic fibrosis led to the development of ivacaftor as a targeted drug for patients with cystic fibrosis [8–10]. Similarly, the success of the ABL1 kinase inhibitor imatinib for chronic myeloid leukaemia provided a clear platform for the field of oncology to move towards application of molecular classification and a focus on genetic strategies for cancer diagnosis and therapeutics [8, 11, 12]. Progress is also now being made in other disease areas—for instance, in the field of cardiology [13] and ischaemic stroke [14].

Parallel to the progress made in precision medicine is the rapid accumulation of administrative, healthcare, and public health data, particularly through electronic health records (EHRs) resulting from clinical encounters, health insurance claims, and medication prescription databases. Beyond traditional data capturing approaches, the rapid developments in technology are also making it possible for important health data to be captured through personal devices, such as mobile phones, wearable devices, activity monitor units, and other emerging technologies that can be used to collect personal data in real time. These developments have greatly enriched the healthcare data space, and this, coupled with increasing capabilities in processing, linking, and analysing these disparate data sources, is opening up new possibilities to utilise data to improve human health [15, 16]. Through advances in high throughput computing and predictive algorithms, machine learning is providing a platform to develop relevant computational algorithms (e.g. decision trees and nested analytic structures) that enable drawing inferences from raw data, in real time, without the need for human input [15, 16].

One of the key foci of precision medicine lies in redefining disease pathogenesis, particularly at the genomic and genetic levels [8, 17, 18]. There has in contrast been far less progress in translating these insights into routine healthcare processes that optimise therapeutic and preventive strategies [8, 19]. Clearly, massive genomic data with potential to improve healthcare decision making are continuously being generated, but the usefulness of such data cannot truly be appreciated until they are successfully integrated into the healthcare system in order to improve human health [8, 19, 20]. Scientific breakthroughs remain incomplete until they are successfully, routinely implemented in clinical settings [20]. Precision medicine has thus far lacked the tools to close the loop from genomic discovery to clinical application [20]. This missing link can be filled by a thoughtful synergy of precision medicine and the principles of learning health systems (LHSs) [20]. LHSs, as will be described in further detail below, invoke as a fundamental precept cyclical processes that convert data to knowledge, bring knowledge to practice, and return the results of implementation into new data and insights, which feed subsequent iterations of the cycle.

In this opinion piece, we use asthma, which is now recognised as being an umbrella term for a heterogeneous group of related conditions, as an exemplar to illustrate the potential synergistic relationship between precision medicine and LHS in improving healthcare processes and clinical decision making. We argue that by capitalising on the underpinning ingredients of implementation science, the approaches endemic to precision medicine and LHS can be successfully integrated in order to improve healthcare and support tailored clinical decision making to the individual patient. In addition, we discuss some of the emerging underpinning issues that need to be harnessed in achieving a synergistic integration of precision medicine and LHSs. These include, but are not limited to, identifying and accessing relevant data sets that need to be accessible for analysis; advancing current electronic data capture systems to accommodate the full range of relevant outcome data; achieving data standardisation and harmonisation; advancing computational capabilities to meaningfully interrogate and analyse these disparate data sets; implementing methods for systematic management and feedback of knowledge created from data analytics; and creating governance mechanisms that allow for secure, trustworthy use and repeated reuse of these data.

## Definition of concepts and transformational goals

### Learning health systems

The LHS focusses on approaches to capture data from clinical encounters and other health-related events, analyse the data to generate new knowledge, and then apply this knowledge to continuously inform and improve health decision making and practice [21–24]. This in turn requires policies, infrastructure, and governance mechanisms to support data-driven health learning and improvement [20, 21]. The LHS capitalises on the advances made in computational science in order to develop algorithms to interrogate health data, interpret these data, and then feed back the gained knowledge to the healthcare systems in order to improve the quality and safety of care [21–24]. The LHS allows identification of at-risk patients, enhances stratification of the population according to different risk profiles, provides decision support tools for clinicians, and provides a platform and infrastructure for facilitating the undertaking of clinical trials [20–24]. More specifically, within the context of clinical trials, the LHS infrastructures are now being used to support efficient recruitment, assess eligibility considerations, undertake point-of-care randomisation, assess outcomes, and enhance long-term follow-up [25–28]. These benefits are not confined to clinical trials; rather, the LHS infrastructure can be used to address a broad array of health problems, as is discussed in detail elsewhere [29].

**Fig. 1:**
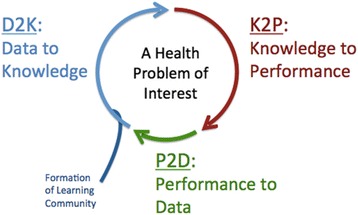
Framework for a learning health system. Adapted from Friedman et al. *Yearb Med Inform*. 2017;26:16–23 [57] with copyright permission granted by the Publisher

As illustrated in Fig. 1, the LHS can be viewed as a cyclical process undertaken by a multi-stakeholder community sharing interest in solving a particular health-related problem. Each cycle begins with conversion of data to knowledge (D2K), followed by application of this acquired new knowledge to transform practice (K2P). The capture of practice changes and the consequences of these changes generate new data, complete the cycle, and initiate the next iteration. Successive iterations of the cycle aim to continue to identify best practices and improve outcomes. The LHS extends principles of continuous quality improvement through the inclusion of governance and infrastructure that enables system improvement to occur with economies of scope and scale [30, 31]. A socio-technical infrastructure also enables the LHS to function with economies of scale and scope. By supporting multiple learning cycles with services, including policy and technology, the cost of executing *N* learning cycles is far less than *N* times the cost of executing one cycle [32]. Because the infrastructure provides services that transcend biomedical domains—that is, the infrastructure supporting an asthma-oriented LHS provides the same services as a cancer-oriented LHS—learning cycles can be directed at any health problem.

One real-life example of the LHS framework is the PINCER (pharmacist-led information technology intervention) trial, which aimed to prevent and correct medication errors in general practice [33]. General practice clinical systems were searched using computerised prescribing safety indicators to identify patients at risk of medication errors on the basis of their prescriptions. With pharmacist support, the identified errors were acted upon and corrected. The system was implemented by an expert team, who used structured activities, in the form of e.g. education, feedback, and opportunities for shared learning, to engage clinicians and pharmacy teams to effect the targeted improvements. Improvements were then measured using anonymised routinely recorded data from general practices collected at three monthly time points. Using appealing illustrative statistics and graphs, feedback was then provided back to the Clinical Commissioning Group and general practices on a continuous basis to assess their performance and enhance continuous improvement (https://sapc.ac.uk/conference/2017/abstract/implementing-pincer-intervention-east-midlands-reduce-prescribing-errors). A key feature of the LHS approach illustrated by this work is the multi-stakeholder ’learning community’.

Additionally, within the context of asthma, we are currently implementing a prototype LHS in Scotland, which has been developed with relevant stakeholders (i.e. patient representatives, clinicians, industry, charity, policymakers, and academics). We are interrogating the EHRs of sampled general practices across Scotland in order to evaluate each general practice’s performance against national care benchmarks for asthma [34]. We plan to create a continuous infrastructure with a feedback (K2P) mechanism that will allow stakeholders to undertake real-time monitoring of the progress being made across important indicators of asthma care at the general practice level. Through linkage of the anonymised clinical data to other demographic, social, and environmental data sets, patients at risk of asthma attacks (structured by various population segments such as gender, age groups, ethnicity, socio-economic status, etc.) will be identified. Findings will then be fed back to clinicians using novel visualisation tools that will allow tailored, actionable decisions to improve asthma control and reduce exacerbations (https://clinicaltrials.gov/ct2/show/NCT03000491).

### Precision medicine

The report of the Precision Medicine Initiative Working Group to the Advisory Committee of the National Institute of Health (NIH) defined precision medicine as “an approach to disease treatment and prevention that seeks to maximise effectiveness by taking into account individual variability in genes, environment, and lifestyle” [17]. The overarching goal has been described as providing a clearer understanding of the development and expression of disease through a “precise delineation of the molecular, environmental, behavioural, and other factors that contribute to health and disease” in order to enhance more targeted diagnosis, treatment, and prevention strategies [8, 17, 18]. Whilst precision medicine has so far been centred around the use of genomic data to achieve individualised clinical decision making, the P4 (i.e. predictive, personalised, preventive, and participatory) paradigm, often linked to precision medicine, highlights the need to take a more holistic systems approach in order to support personalised healthcare decision making [35–39]. This extended paradigm is dependent on integration of genetic, phenotypic, administrative, and sociotypic data. Achieving integration of these data types will require the creation of deeply characterised national longitudinal cohorts that are continuously mined to support delivery of personalised care and to improve care processes [35–39]. Most conceptions of precision medicine leave implicit the knowledge-to-practice processes so clearly called out in the LHS.

## Precision medicine initiatives using asthma as an illustrative example

The concept of ‘one size does not fit all’, fundamental to precision medicine, has been reinforced in the context of asthma, indicating that the traditional understanding of asthma as a single disease element is outdated [40]. Rather, accumulated evidence now shows that asthma is a heterogeneous disease characterised by variability in its pathophysiologic mechanisms (endotypes) and underlying patients’ characteristics (phenotypes) [40–43]. The heterogeneity of asthma also manifests in variability of clinical outcomes that influence treatment options [40–43]. Whilst asthma has been described as a condition with both type 2 and non-type 2 immune responses, it is the type 2 immune response endotypes that have been best characterised [40, 41]. Type 2 immune response endotypes underlie the common atopic asthma phenotypes, characterised by eosinophilic airway inflammation, increase in type 2 cytokine levels, and aspirin-exacerbated respiratory responses [40]. Subendotypes of type 2 immune response endotype include the interleukin (IL)-5-high, IL-13-high, and immunoglobulin E (IgE)-high endotypes [40].

From progress made in genomic profiling, it has been demonstrated that subtypes of asthma are related to distinct molecular perturbations. Tailoring treatment to different asthma subtypes/endotypes may improve clinical outcomes of asthma and reduce the risk of adverse events [40, 44, 45]. For example, patients with type 2 immune response asthma endotypes appear to respond to treatment targeting the IL-5, IL-13, and IgE-mediated pathophysiological pathways [40]. Several asthma biomarkers have also been identified and are currently being used to target treatment options in relation to type 2 immune-related inflammation. For example, blood eosinophilia has been shown as a targeted biomarker linked to corticosteroids, as well as anti-IL-4-, anti-IL-13-, and anti-IL-5-targeted treatment; sputum eosinophil levels are also used as biomarkers for predicting treatment responses to inhaled steroids and anti-IL-13 and anti-IL-5 treatment; whilst serum periostin levels are linked to anti-IL-13 therapy, periostin present in bronchial tissue has been shown as a biomarker for eosinophilic airway inflammation [40]. Several other biomarkers have also been shown to predict treatment response for patients with type 2 immune response-related asthma inflammation. However, whilst most asthma biomarkers are being used for research purposes, their clinical application remains uncertain, as they are yet to be validated and qualified—i.e. linking many available biomarkers with clear clinical endpoints is still at various stages of development [40].

**Fig. 2:**
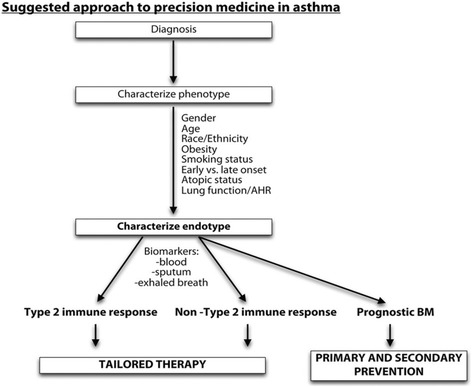
PRACTALL suggested steps for implementing precision medicine in asthma. *AHR* airway hyper-responsiveness, *BM* biomarkers. Reproduced from Muraro et al. *J Allergy Clin Immunol*. 2016;137:1347–58 [40]

In considering the application of precision medicine to achieve personalised therapy for patients with asthma, the PRACTALL collaboration (joint expert group of the European Academy of Allergy and Clinical Immunology [EAACI] and the American Academy of Allergy, Asthma & Immunology [AAAAI]) outlined a three-step approach (Fig. 2) [40]. As a first step, there is a need to correctly establish and verify the diagnosis of asthma, including adequate treatment of any co-morbidity. This should be followed by the establishment of the underlying asthma phenotype based on the physical characteristics of the patients. Third, the underlying asthma endotype needs to be ascertained, as a clear understanding of the pathophysiological mechanism of a particular endotype is crucial for delivering targeted personalised treatment. Finally, there is a need to validate known biomarkers that may be related to asthma severity and clinical outcomes, as this will be essential for further development of primary and secondary asthma prevention strategies [36]. Overall, this model leaves the K2P framework (see Fig. 1) implicit and, as such, this example does not reflect the full potential of an integration of precision medicine and LHS.

## Link between precision medicine and LHS: theoretical framework

Whilst the primary focus of precision medicine has thus far been on the discovery side of science, there is also a need to pay attention to how to translate the discoveries being made into clinical practice. This should be followed by efforts to systematically evaluate outcomes at an individual patient level and iterate the process, as needed, with the overall goal of ensuring that the discoveries made at the genomic level are used to improve clinical care for individual patients (see Fig. 1). The National Academy of Medicine, in its report based on the Roundtable on Translating Genomic-Based Research for Health, provided a springboard for discussion on the strategies of translating genomic breakthroughs into clinical practice, emphasising the need to capitalise on the underpinning theoretical frameworks in innovation sciences [46]. The pathways to translating research findings to the clinical setting are complex, often inhibited by known and unknown barriers. It is insufficient to know whether a particular intervention works in controlled environments. Health improvement requires that the intervention works and can be cost-effectively delivered in real-world settings [20].

The LHS can provide the conceptual framework and infrastructure platform to ensure the real-time iterative application of breakthroughs in precision medicine to a real-world healthcare setting, but the pathway to achieving this may not be straightforward [20]. Chambers and colleagues have suggested a theoretical framework through which precision medicine and LHS can be synergistically integrated, emphasising that implementation science may provide the missing link to this complex convergence [20]. Implementation science has been defined as “the scientific study of methods to promote the systematic uptake of research findings and other evidence-based practices into routine practice, and, hence, to improve the quality and effectiveness of health services and care” [37]. Healthcare delivery is complex, with a number of barriers inhibiting the emergence of high efficiency health systems. Implementation science therefore focusses on identifying and modifying potential factors, both individual and policy factors, that tend to influence the uptake and application of research findings into real-world clinical practice [44–50].

**Fig. 3:**
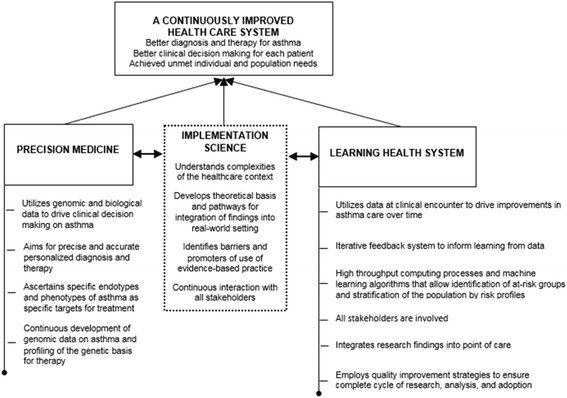
A framework for a synergy between precision medicine and LHS: the role of implementation science as a catalyst of the link. Reproduced with permission from Chambers et al. *JAMA*. 2016. 10;315(18):1941-2 [58] Copyright© (2016) American Medical Association. All rights reserved

We have built on this framework to propose the model depicted in Fig. 3 (illustrated for asthma). The proposed framework indicates that the ultimate goal of a synergy between precision medicine and LHS is to achieve a continuously improved health system in which precision medicine is considered a core ingredient of a learning health cycle, which includes development of improved and better targeted diagnosis and therapy, allowing for better decision making for each patient presenting at the clinical care level. Evidence developed using precision medicine approaches, for example, the establishment of treatment options targeting specific subendotypes or phenotypes of asthma, therefore needs to be assessed on whether such discoveries can be translated to improve clinical outcomes of asthma and on whether it is feasible to identify subgroups of asthma patients for whom such a potential treatment option can be applied. Moving evidence from precision medicine based on genetic targets will require linking genomic data to clinical electronic health data [51]. This process has ethical, policy, economic, and technical dimensions that need to be addressed, and are illustrative of the range of challenges that stand between the current state of health systems and their potential to become learning systems capable of realising the full potential of precision medicine. One important solution is the establishment of Safe Havens that provide a secure infrastructure for undertaking data-intensive research and clinical care. Concerning privacy and data safety, Taitsman and colleagues recently identified three levels of safeguards: physical, electronic, and human capital [52]. At the physical level, patient data should be treated with the utmost level of confidentiality including making patient-physician consultation as private as possible and enabling a secure storage of all patient data. At the electronic level, access to patient data should be user-authenticated: systems should be protected with appropriate firewalls, antivirus programs, and necessary encryptions; and storage hardware should be made as secure as possible. At the human capital level, hiring of database personnel should follow careful vetting and background checks; personnel should undertake appropriate training on information governance, data security, and patient privacy, and they should be equipped with necessary data sharing and security protocols [52].

The framework embodied in Fig. 3 highlights the strengths of implementation science, which can serve as a catalyst of achieving a synergy between precision medicine and LHS. Through implementation science, fundamental elements of healthcare settings, the cultures that underpin them, and the specifics of each context can be identified, analysed, and appropriately managed. By involving and partnering with all relevant stakeholders, implementation science capitalises on core strategies (planning, education, financing, restructuring, quality management, and awareness of policy contexts) to achieve its goal and to support a successful link between precision medicine and LHS, so that promising findings generated from genomic data can be successfully translated into real-world settings to improve asthma care. This synthesis, if achieved, will realise a paradigm shift resulting in, among other changes, the perception of genomic data and information derived from them as part of data supporting routine care. Such a paradigm shift requires continuous education of patients, healthcare providers, and the general population on the emerging developments in our understanding of disease and the impact they may have on routine clinical practice [13, 19].

## Underpinning infrastructure and strategies for scaling up

Achieving the goal of precision medicine that has relevance to day-to-day healthcare processes and decision making, through the application of LHS principles and with implementation science as a bridge, will require a shared digital infrastructure to both promote personalisation and continuously improve the overall system [35, 53]. One of the first fundamental challenges is to identify data assets (i.e. genomic, clinical, phenotypic, sociotypic, and environmental data sets) that are relevant to the health problems being addressed [35, 53]. From the healthcare system perspective, there is a need to enhance current electronic data systems in ways that enable them to capture and integrate multiple sources of outcome data, such as administrative, social, and patient-reported outcomes/experiences (PROMS/PREMS) data [35, 53]. The sources and nature of these data sets are usually disparate; hence, there is a need for establishment of frameworks for data standardisation, harmonisation, transformation, and linkage [53]. Such tools and analysis platforms will benefit from being transferrable across contexts, adaptable to multiple computational platforms, and open source in order to allow for further developmental inputs from all stakeholders, which will be essential in realising their full potential in advancing the field [53]. These tools should also support meaningful visualisation of data in ways that will allow benchmarking and assessing personal risk.

Approaches to analysis of observational data need to be robust, with case-mix adjustments and propensity score techniques; whilst these have usually been achieved through conventional frequentist statistical approaches, where possible, they should be complemented by artificial intelligence-based approaches (e.g. machine learning) that provide meaningful learning from non-dimensional complex data sets. These data systems and the tools needed to continuously interrogate them must be complemented by ’knowledge to practice’ infrastructures (e.g. Apervita [https://apervita.com/], Semedy [http://www.semedy.com/]) that engage organisations, healthcare professionals, and patients actively in health and healthcare improvement [35, 53]. These strategies will thrive in a relevant policy-enabling context: the issues of data ownership, security, privacy and anonymity, and data sharing need to be carefully outlined, with the contribution of all stakeholders [53, 54]. Our increasing understanding of disease process and health now clearly reveal the complex inter-relationships between genetic, physiological, social, behavioural, and health system determinants. Although inherently challenging, particularly as the volume and complexity of the available data increase, there is a need to develop data processing and analytical capabilities that will help bring together various relevant data sets, interrogate these data sets to uncover the underlying interactions, and then integrate the findings into routine clinical care [55, 56].

## Conclusions

The potential of precision medicine in individualising healthcare decision making will not be fully realised until emerging breakthroughs are successfully integrated into routine clinical care. We have proposed a framework of integrating accumulating genomic data into the clinical encounter in a way that allows for a continuous learning health cycle and improvement of the quality of care. We have emphasised the role of implementation science as an important catalyst in linking precision medicine to the healthcare system. Through partnership with all relevant stakeholders, implementation science needs to support the translating of findings generated from precision medicine into real-world healthcare settings. Furthermore, in order to enable scale-up of an integrated system, the underlying digital infrastructure needs to promote personalisation and continuously improve the overall system need to be shared: this includes identification of all relevant data; advancement of current electronic data capture systems to accommodate other sources of outcome data (e.g. administrative and social data); establishment of frameworks for data standardisation and harmonisation; creation of frameworks for effective linkage of the various data sets in a secure and transferrable way; advancement of the computational capabilities to meaningfully interrogate and analyse these data; development of decision support systems that will enhance active participation of organisations, healthcare professionals, and patients in the healthcare processes; and finally the need to create a policy-enabling context that defines and sets necessary limits for data ownership, security, privacy, and data sharing.

## Abbreviations

AAAAI: American academy of allergy, asthma & immunology
CFTR: Cystic fibrosis transmembrane conductance regular
D2K: Data to knowledge
EAACI: European academy of allergy and clinical immunology
EHR: Electronic health record
K2P: Knowledge to practice
LHS: Learning health system
NIH: National institute of health
P2D: Performance to data
P4: Predictive, personalised, preventive, and participatory
PINCER: Pharmacist-led information technology intervention
PRACTALL: Precision medicine in patients with allergic diseases
PREMS: Patient-reported experiences
PROMS: Patient-reported outcomes
